# Target Recognition Triggered Split DNAzyme based Colorimetric Assay for Direct and Sensitive Methicillin-Resistance Analysis of *Staphylococcus aureus*

**DOI:** 10.4014/jmb.2404.04012

**Published:** 2024-04-19

**Authors:** Jin Xu, Dandan Jin, Zhengwei Wang

**Affiliations:** Department of Anesthesiology, People’s Hospital of Chongqing Liang Jiang New Area, Chongqing 401121, P.R. China

**Keywords:** MRSA, aptamer, split DNAzyme, G-quadruplex

## Abstract

The accurate and rapid detection of methicillin-resistant *Staphylococcus aureus* (MRSA) holds significant clinical importance. This work presents a new method for detecting methicillin-resistant *Staphylococcus aureus* (*S. aureus*) in clinical samples. The method uses an aptamer-based colorimetric assay that combines a recognizing probe to identify the target and split DNAzyme to amplify the signal, resulting in a highly sensitive and direct analysis of methicillin-resistance. The identification of the PBP2a protein on the membrane of *S. aureus* in clinical samples leads to the allosterism of the recognizing probe, and thus provides a template for the proximity ligation of split DNAzyme. The proximity ligation of split DNAzyme forms an intact DNAzyme to identify the loop section in the L probe and generates a nicking site to release the loop sequence (“3” and “4” fragments). The “3” and “4” fragments forms an intact sequence to induce the catalytic hairpin assembly, exposing the G-rich section. The released the G-rich sequence of LR probe induces the formation of G-quadruplex–hemin DNAzyme as a colorimetric signal readout. The absorption intensity demonstrated a strong linear association with the logarithm of the *S. aureus* concentration across a wide range of 5 orders of magnitude dynamic range under the optimized experimental parameters. The limit of detection was calculated to be 23 CFU/ml and the method showed high selectivity for MRSA.

## Introduction

The infection in patients undergoing surgical treatment is mostly a nosocomial infection caused by bacteria, which not only exacerbates the patient’s suffering during treatment but also poses a significant cost burden. *Staphylococcus aureus* is a prevalent pathogen that is often acquired in hospitals [1, 2]. Nevertheless, the escalation of *S. aureus*’s ability to resist methicillin has resulted in inadequate treatment of surgical infections and has a severe impact on patients’ postoperative recovery [3, 4]. Hence, conducting precise investigation of antibiotic resistance in *S. aureus* holds immense importance in ensuring optimal surgical recovery and patient care. Efficient identification of methicillin-resistant potential of *S. aureus* directly from clinical specimens is crucial for gathering information regarding antimicrobial drug susceptibility, and thus guiding antimicrobial therapy and ultimately lower mortality rates [5-7]. The *mecA* gene is responsible for methicillin resistance by encoding for penicillin-binding protein 2a (PBP2a), which acts as a replacement for other PBPs in the process of cross-linking peptidoglycan chains [8].

The rapid and precise diagnosis of methicillin-resistant *Staphylococcus aureus* (MRSA) is essential for appropriate infection management. Antimicrobial susceptibility testing is commonly used as the standard method for detecting MRSA due to its simplicity [9, 10]. However, its application is hindered because it is time-consuming and is possible for false-negative results. The utilization of a costly antibody poses a hindrance to the implementation of immunological techniques, despite its sufficient level of specificity. Nucleic acid-based strategies offer greater advantages compared to other methods due to their ability to identify antibiotic resistance genes, such as the *mecA* gene [11]. In general, polymerase chain reaction (PCR) and other isothermal amplification technologies, such as loop-mediated isothermal amplification and nucleic acid sequence-based amplification, have sufficient sensitivity [12-14]. However, the PCR-based method requires advanced equipment and accurate temperature cycling, whereas isothermal amplification methods are not available to detect a single nucleotide. In addition, the cell lysis and gene extraction is essential for these nucleic acids based approaches, which greatly complicated the detection procedures [5]. Therefore, the development of a sensitive, specific, rapid, and direct strategy for methicillin resistance analysis in *S. aureus* is crucial.

Aptamers, referred to as chemical antibodies, are single-stranded DNA or RNA molecules that exhibit a strong and particular binding affinity for a diverse array of molecule [15]. These aptamers have found extensive application in the detection of the PBP2a protein, owing to their exceptional specificity and affinity towards the target molecules [16, 17]. Aptamers, in comparison to typical antibodies, exhibit greater stability and are more readily prepared and modified [18, 19]. This renders them viable substitutes for antibodies as recognition agents in *S. aureus* sensors. Aptamer-based systems have been devised employing various detection techniques such as fluorescence [20, 21], colorimetry [16, 22], and electrochemistry to identify intact cells of *S. aureus*. Nevertheless, the utilization of an aptamer that specifically targets PBP2a, a membrane protein associated with penicillin resistance in *S. aureus* is infrequently reported. Colorimetric sensing is an analytical technique that detects and measures analytes of interest by observing changes in color or absorbance of a chemical species [23]. It has gained significant attention for its applications in point-of-care testing (POCT). Conventional colorimetric methods typically necessitate costly equipment and intricate sample preparation, hence restricting their availability in settings with limited resources [24]. As a result, there is still a high demand for novel, simple, and highly sensitive techniques to directly analyze penicillin resistance in *S. aureus* for the purpose of detecting infections.

**Fig. 1:**
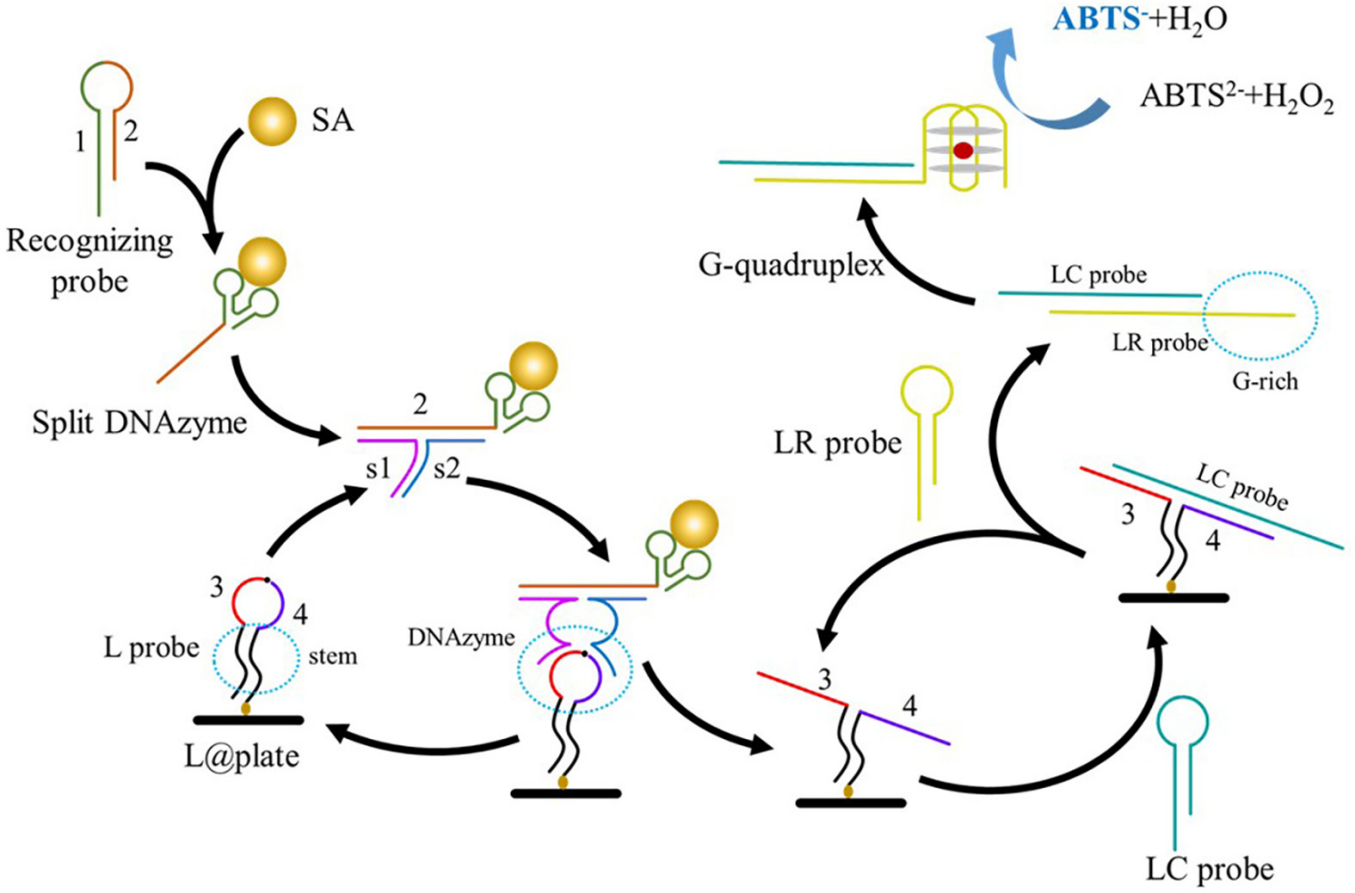
The working mechanism of the proposed method for direct methicillin resistance analysis.

In the current study, an aptamer-based colorimetric biosensor was developed for the rapid, ultrasensitive, and visual detection of MRSA using recognizing probe and the split DNAzyme system. In this sensing strategy, an aptamer, which is integrated in the recognizing probe with loop-stem structure, was employed to specifically bind with PBP2a protein on the surface of *S. aureus* (Fig. 1). In the present of target bacteir with PBP2a, the aptamer (“1”) in the recognizing probe binds with the PBP2a protein and releases the “2” section to induce the subsequent signal amplification. In detail, the toeholds of the split DNAzymes (s1 and s2) bind with the released “2” section. The s1/s2/“2” complex forms an active secondary DNAzyme structure with s1 and s2 sequence hybridize with the loop section of the L probe. Upon the addition of metal ion, the assembled DNAzyme generates a nicking site in the loop section through cleaves the “rA” point. Based on this the “3” and “4” sections are released to medicate the catalytic hairpin amplification. Specifically, the “3” and “4” unfolds the LC probe and initiates the assembly between LC probe and LR probe, releasing the G-rich section in LP probe. Moreover, the L@plate is liberated to bind with a next LC probe, forming a signal cycle. The released G-rich section forms a Horseradish peroxidase (HRP)-mimicking DNAzyme to induce the color reaction. The amount of PBP2a protein was proportional to the UV-vis absorbance value of the oxidation product of 2,2'-azino-bis(3-ethylbenzothiazoline-6-sulfonic acid) (ABTS^2–^) catalyzed by the produced HRP-DNAzyme, and the accompanying color change was visible to the naked eye.

## Materials and Methods

### Materials and Methods

The sequences used in this method were synthesized and purified by Sangon Biotech (China). The detail of the sequences used in this research is shown in Table S1. The bacteria, including the methicillin-resistant *S. aureus* (MRSA) N315, methicillin-sensitive *Staphylococcus aureus* (MSSA) CCTCC AB91118, *Escherichia. coli* O157:H7 (*E coli*) NCTC 129007, and *Staphylococcus epidermidis* (*S. epidermidis*) ATCC 12228 were kindly provided by the central laboratory. The reagents for color reaction, including 2,2'-Azino-bis(3-ethylbenzothiazoline-6-sulfonic acid) (ABTS^2–^) and 4-(2-hydroxyethyl)piperazine-1-ethanesulfonic acid sodium salt (HEPES), were obtained from Sigma-Aldrich (USA). Hemin and H_2_O_2_ were provided by Sangon Biotechnology Co., Ltd., (China). All other chemicals were of analytical grade and used without further purification. Ultrapure water (resistance > 18 MΩ) was used throughout the experiments.

### Detection Procedure of the Method

300 nM of the recognizing probe (10 μl) and various concentrations of MRSA (10 μl) were mixed in a tube containing 30 μl of PBS buffer. The mixture was incubated at room temperature for 30 min, followed by the mixing with the L probe@plate. Afterwards, 10 μL of the s1 and s2 sequences (100 nM, respectively) were added to the plate. The mixture was incubated at room temperature for 30 min. afterwards, the liquid supernatant was removed from the plate well, and the plate was washed for 3 times with PBS buffer solution. 10 μl of the LC and LR probe (100 nM, respectively), 20 μl of PBS buffer solution were added to the palte for the color reaction. Finally, 1 μl of hemin (20 μM, prepared in DMSO), 9 μl of 10 × HEPES (250 mM HEPES, 2 M NaCl, 100 mM KCl, and 0.5%Triton X-100, pH 5.2), and a sufficient amount of H_2_O were added to form a resultant solution of 90 μl, and then incubated for 30 min at 37°C. UV-vis absorption spectra were recorded at a fixed reaction time of 10 min after the addition of 10 μl of 4 mM ABTS^2–^ and 10 μl of 400 mM H_2_O_2_ to the solutions.

## Results and Discussion

### Assembly of Recognizing Probe and Feasibility Analysis

**Fig. 2:**
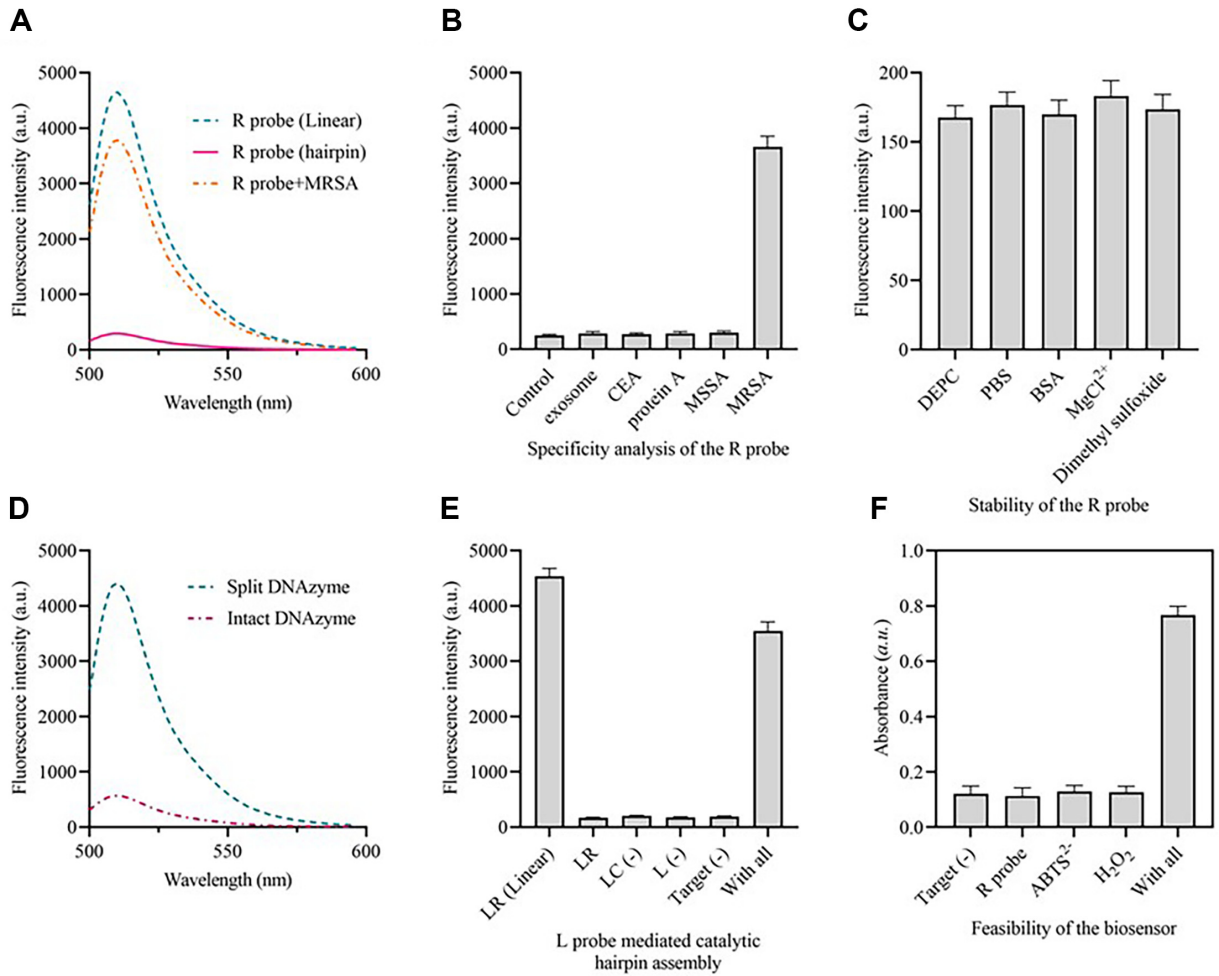
Feasibility analysis of the proposed method. (**A**) Fluorescence spectrum of the FAM labeled recognizing probe when MRSA exists or not. (**B**) Fluorescence intensities of the recognizing probe for target and interfering molecules detection. (**C**) Fluorescence intensities of the recognizing probe under different experimental conditions. (**D**) Fluorescence spectrum of the FAM labeled split DNAzyme before and after proximity ligation of split DNAzyme. (**E**) Fluorescence intensities of the FAM labeled LR probe during the catalytic hairpin assembly process. (**F**) Absorbance of the approach during MRSA detection. Inserted is the visible absorbance changes. *n* = 3 technical duplicates.

A fluorescent assay was conducted to determine if the developed recognizing probe could selectively identify the target bacterium and subsequently mediate the proximity ligation of split DNAzyme. Fig. 2A demonstrates that the fluorescence intensity at 510 nm of the recognizing probe (R probe) was noticeably greater when incubated with the target compared to probes without the target. This indicates that the presence of bacteria can trigger the allosterism of the recognizing probe. To investigate the potential unfolding of a recognizing probe, various interfering molecules, including exosomes, protein A, MSSA (methicillin-sensitive *Staphylococcus aureus*, CCTCC AB91118), and CEA, were incubated with the recognizing probe. Based on the result, we have observed slight increases in fluorescence when there were interfering molecules present, compared to the control group. This suggests that the interfering molecules had minimal impact on the recognition of the probes (Fig. 2B). Furthermore, the probe demonstrated exceptional stability even in a complex environment containing diethyl pyrocarbonate (DEPC), BSA, MgCl^2+^, and dimethyl sulfoxide, as depicted in Fig. 2C. Subsequently, we examined if the recognizing probe may facilitate the proximity ligation of the split-DNAzyme (s1 and s2). Ultimately, we noticed a substantial reduction in fluorescence when the target was present in the sensing system, indicating the effective proximity ligation of the split-DNAzyme (Fig. 2D). Fluorescence assays were employed to confirm the catalytic hairpin construction triggered by DNAzyme-treated L probe, wherein the fluorescent dye and quenching groups were labeled at both ends of the LR probe. The findings are displayed in Fig. 2E. In the presence of the target, a notable increase in the fluorescence signal is obtained, suggesting that the LR probe has been activated and the fluorescence signal has been released. However, the absence of the LC probe and L probe did not result in any noticeable increase in fluorescence. The viability of the entire procedure was confirmed by monitoring the alteration in color within the 96-well plate. The findings are displayed in Fig. 2F, where noticeable alterations in color and absorbance are detected exclusively in the presence of the target.

### Experimental Parameter Optimization

**Fig. 3:**
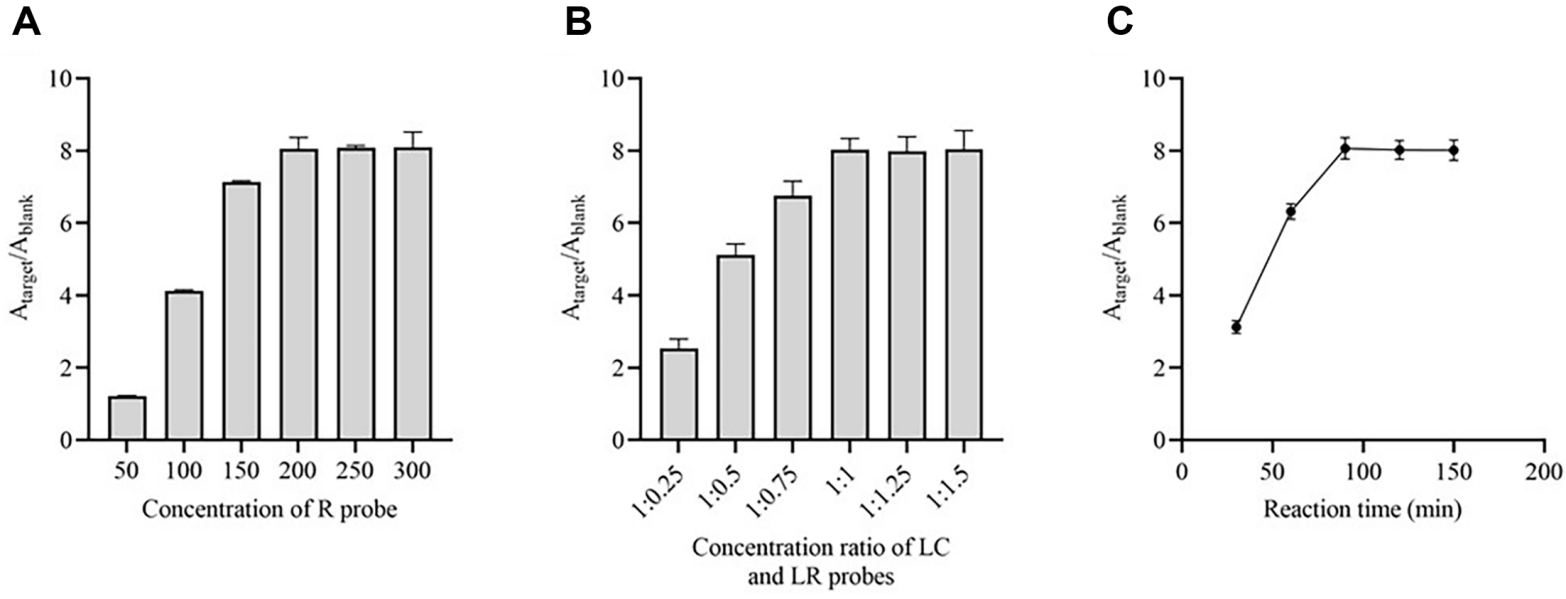
Optimization of experimental parameters. Absorbance ratio of the approach when detecting MRSA with different R probe concentrations (**A**) concentration ratio of LC and LR probes (**B**) reaction time (**C**). *n* = 3 technical duplicates.

Prior to assessing the detection performance, we refined the experimental parameters of the approach, such as the concentration of the recognizing probe, the ratio of LC and LR probes, and the duration of the reactions. The A_target_/A_blank_, which is the ratio of the absorbance when target existed (A_target_) or not (A_blank_), is used to compare the detection performance of the method. In the studies examining the concentration of recognizing probes, the absorbance values obtained showed a gradual increase as the concentration of recognizing probes increased from 50 to 200 nM. However, no further increase in absorbance values was found at higher concentrations (Fig. 3A). Thus, the concentration of the recognizing probes was determined to be 200 nM. Furthermore, the proportion of LC and LR probes and the duration of the reaction were established as 1:1 (Fig. 3B) and 90 min (Fig. 3C), respectively.

### Sensitive and Selectivity Detection of Methicillin Resistance *S. aureus*

**Fig. 4:**
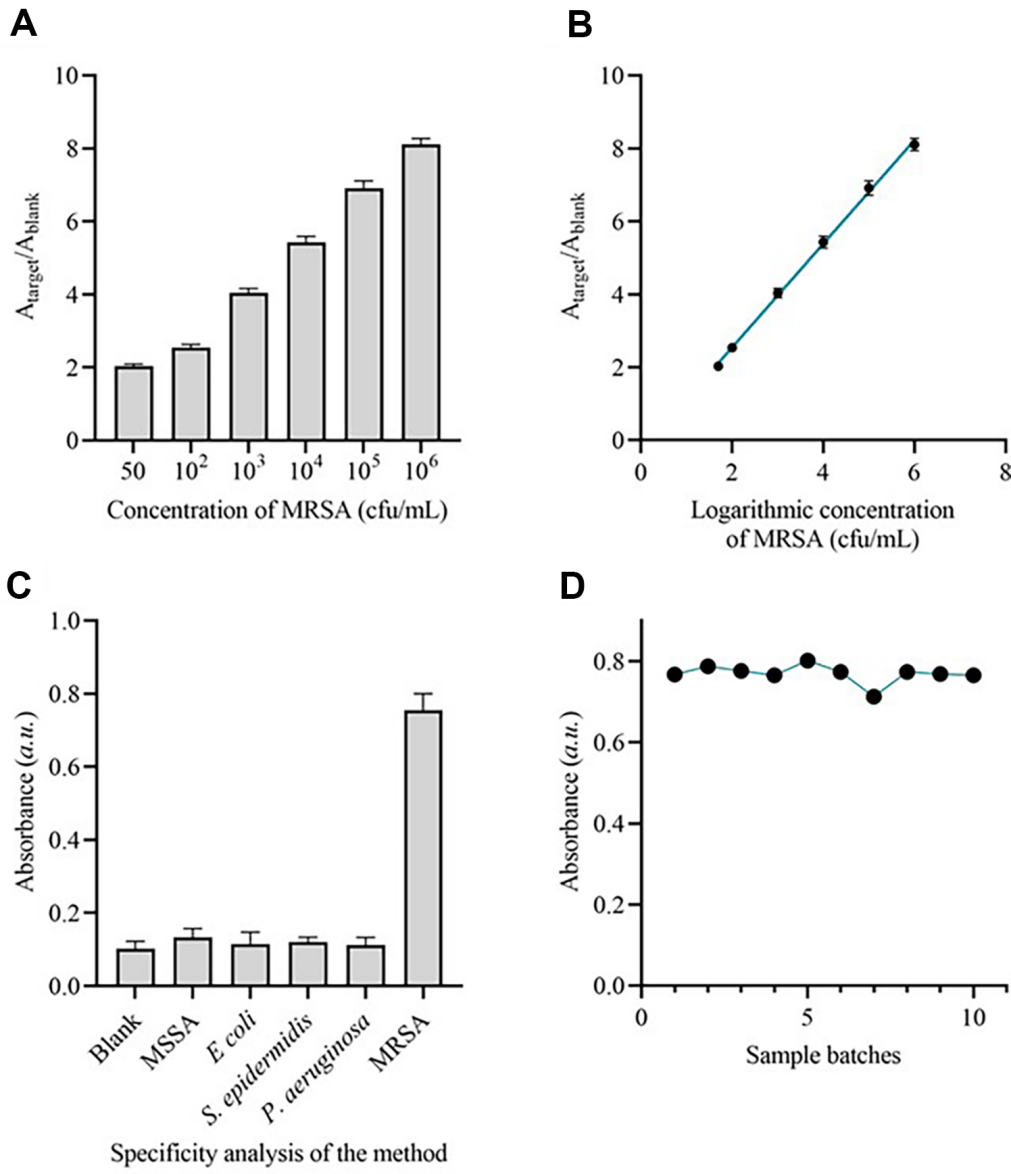
Analytical performance of the method. (**A**) Absorbance ratio of the approach when detecting MRSA with different concentrations. (**B**) Correlation between the absorbance ratio and the concentration of MRSA. (**C**) Absorbance of the approach when detecting MRSA and interfering bacteria. *n* = 3 technical duplicates. (**D**) Absorbance of the approach when detecting MRSA from 10 sample duplicates.

The suggested colorimetric method was used to detect various quantities of MRSA N315 in PBS under optimized experimental conditions. To evaluate the capability of the established method for sensitive detection of MRSA, a range of target concentrations were prepared and examined. Fig. 4A displays the UV-vis absorption signals for different concentrations of the target. The absorbance consistently increased as the target concentrations ranged from 50 CFU/ml to 10^6^ CFU/ml. Fig. 4B illustrates the linear relationship between the absorption intensities and the logarithm of the target concentration. The range of concentrations considered is from 50 CFU/ml to 106 CFU/ml. The linear correlation coefficient for this relationship is 0.9932. Based on the 3σ rule, the estimated detection limit is 23 CFU/ml, which above the detection limit of current approaches.

The detection performance of a biosensor’s performance relies heavily on its selectivity. We employed three interfering bacteria to examine the discerning detection capability of the approach. The absorption intensities can clearly be distinguished of MRSA from MSSA, *E. coli*, *S. epidermidis*, and *P aeruginosa*. Observably, the peak intensity experiences a reduction of around 82% when there are interfering bacteria present, in comparison to the target at the identical concentration (Fig. 4C). In order to assess the repeatability of the system, the 1.0 × 10^5^ CFU/ml of MRSA was detected 10 sample duplicates. The coefficients of variation (CV) were 4.12% (Fig. 4D), suggesting that the detection system had satisfactory repeatability.

### Analytical Performance of the Approach

**Fig. 5:**
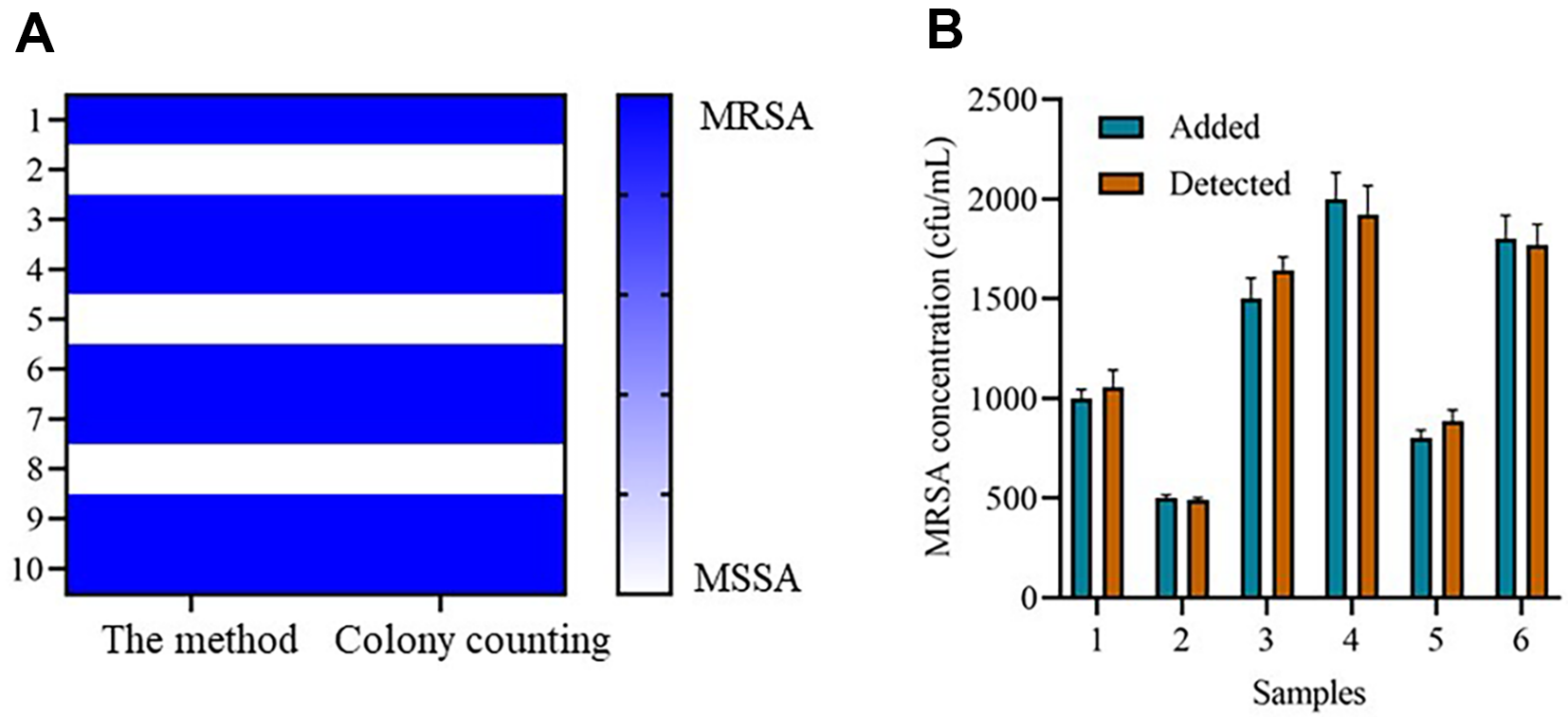
Clinical application of the method. (**A**) MRSA positive sample reported by the method and colony counting method. (**B**) MRSA concentration calculated by the method from samples added with different concentrations of MRSA.

Target bacterial strains and MSSA strains were analyzed by colony counting method and the proposed method to evaluate the detection effect. The colony counting test results showed that 10 samples contained bacteria with methicillin resistance, which proved that 7 strains were MRSA, 3 strains were MSSA. The results of the proposed detection system also show the same result in all strains of bacteria such as 7 strains detected as MRSA, 3 strains were MSSA (Fig. 5A). The stability of the proposed method was assessed using 10% commercial whole blood supplemented with bacteria. As illustrated in Fig. 5B, by comparing the characteristic peak values, the recovery rates of the MRSA concentration calculated by the method ranged from 98.78% to 102.3%, indicating a high stability of the method in complicated experimental conditions.

## Conclusion

In this study, an aptamer-based colorimetric assay for MRSA detection was constructed based on the high affinity of aptamer to target protein PBP2a. In this method, the PBP2a protein aptamer is integrated in a recognizing probe with a hairpin structure. the specific target recognition leads to the allosterism of the recognizing probe, which provides a template for the proximity ligation of DNAzyme. The assembled DNAzyme cleaves the L probe on plate to induce subsequent signal amplification and color reaction. Eventually, the method exhibits a low limit of detection of 23 CFU/ml and high specificity towards MRSA compared to MSSA with potentials for rapid detection of MRSA in real clinical specimens, such as plasma. In addition, the total time of the method for MRSA detection from the clinical sample preparation to susceptibility result was less than 2 h. In contrast, the conventional susceptibility testing, which includes the use of the automated Vitek 2 system, requires at least 24 h, including 18-24 h for isolating and purifying bacteria from the clinical samples, and an additional 7 h for conducting susceptibility testing. Ultimately, a novel method for rapid and direct MRSA analysis has been created by combining split DNAzyme based signal recycling and ABTS based color reaction. Due to its short reaction time and convenience, this aptamer-based colorimetric assay should be a useful tool for more rapid and accurate detection of MRSA in clinical specimens.

## Supplemental Materials

Supplementary data for this paper are available on-line only at http://jmb.or.kr.
