# Supplementary material for: Target Recognition Triggered Split DNAzyme based Colorimetric Assay for Direct and Sensitive Methicillin-Resistance Analysis of Staphylococcus aureus
Source: J Microbiol Biotechnol. 2024 Apr 19;34(6):1322–7. doi: 10.4014/jmb.2404.04012 (PMC11239412; doi:10.4014/jmb.2404.04012)
Supplement: Supplementary file 1 [file jmb-34-6-1322-supple.pdf]

J. Microbiol. Biotechnol. 2024. 34(6): 1322–1327
https://doi.org/10.4014/jmb.2404.04012

Supplementary Table

Table S1. Oligonucleotide sequences used in this work.

Title	Sequences (5' to 3')
R probe	CCA TCC ACA CTC CGC AAG GGT GCC CCG GGG GG CTG TTC AGC GTG GTG GTG GGA TGC CGT TTT GGT CCT TAG TCT CCG TCG TCG GCT GCC TCT ACA TTA G CTT ATC AGA CTG ATG TTG A CCCCCC GGGGCA CCCTTG
s1	TCAACATCAGTCGGTCGAAATAGTGAGTCGCTC
s2	CATCTCTTCTCCGAGCCTGATAAGCTA
L probe	TTT TTT TTT TTT GTG AGG CAC GTT AGA CCA CTT GAG CGA CTC ACT ATrA GGA AGA GAT G ATC TCC ACA ACT GAA CCT CAC AAA AAA AAA AAA
LC	ACA ACT GAA CAC GTT AGA CCA CTT CCA TCC TCG CAA ATC TCC ACA ACT AAG TGG TCT AAC GTG TTC AGT TGT GGA GAT
LR	GGG TAG GGC GGG TTG GGA TCC TGG GAA ATC TCC ACA ACT GAA CAC GTT AGA CCA CTT AGT TGT GGA GAT TTG CGA GGA TGG AAG TGG TCT AAC
